# Consumption of Farmed Fish, Fed with an Olive-Pomace Enriched Diet, and Its Effect on the Inflammatory, Redox, and Platelet-Activating Factor Enzyme Profile of Apparently Healthy Adults: A Double-Blind Randomized Crossover Trial

**DOI:** 10.3390/foods11142105

**Published:** 2022-07-15

**Authors:** Filio Petsini, Agathi Ntzouvani, Maria Detopoulou, Vasiliki D Papakonstantinou, Nick Kalogeropoulos, Elizabeth Fragopoulou, Tzortzis Nomikos, Meropi D Kontogianni, Smaragdi Antonopoulou

**Affiliations:** 1 Department of Nutrition and Dietetics, School of Health Sciences and Education, Harokopio University,17671 Athens, Greece; petsini.filio@gmail.com (F.P.); agathintz@gmail.com (A.N.); mdetopoulou@gmail.com (M.D.); nickal@hua.gr (N.K.); efragop@hua.gr (E.F.); tnomikos@hua.gr (T.N.); mkont@hua.gr (M.D.K.); 2 Laboratory of Biochemistry, Department of Chemistry, National and Kapodistrian University of Athens, 15771 Athens, Greece; papakonstantinou.v@gmail.com

**Keywords:** platelet activating factor (PAF), PAF enzymes, PAF-CPT, lysoPAF-AT, PAF-AH, LpPLA_2_, inflammation, oxidative stress, enriched fish, olive pomace

## Abstract

A fish-rich diet has a beneficial effect on cardiovascular health. The platelet activating factor (PAF) is involved in the development of atherosclerosis, and in vitro results support the regulating action of bioactive nutrients on PAF metabolism. The purpose of this study is to examine whether the consumption of farmed fish fed with an olive-pomace enriched diet (EF) affects PAF metabolism and the markers of inflammation and oxidative stress compared to the consumption of conventionally fed farmed fish (CF). Thirty apparently healthy adults completed a randomized double-blind crossover trial, during which they consumed both CF and EF twice a week for 8 weeks with a six-week washout period in between. The activities of PAF acetylhydrolase (PAF-AH), lysoPAF acetyltransferase (lysoPAF-AT), DTT-insensitive CDP-choline: 1-alkyl-2-acetyl-sn-glycerol-choline-phosphotransferase (PAF-CPT) in leukocytes, and lipoprotein-associated phospholipase A_2_ (LpPLA_2_) in serum were determined. The quantities of interleukin-6 (IL-6), high sensitivity C-reactive protein (hsCRP), oxidized LDL (ox-LDL), thiobarbituric acid-reactive substances (TBARS), and glutathione peroxidase (GPx), as well as the serum oxidation, were also determined. Both types of fish exerted similar effects as there were no statistically significant differences between the two interventions except for an elevated PAF-CPT and reduced arachidonic acid (AA) in the red blood cell (RBC) membrane lipids after the EF intake.

## 1. Introduction

Cardiovascular health is linked to diet. Unhealthy dietary habits, such as increased salt and fat consumption, have been linked to the development of cardiovascular diseases (CVD). In contrast, following a healthy dietary pattern, such as the Mediterranean dietary pattern, may prevent the emergence of CVD, and reduce the severity of the established disease. Fish, being an important “ingredient” of a healthy diet, has been widely studied for its cardioprotective effects. Studies have linked frequent fish consumption or fish oil supplementation with the amelioration of the biomarkers of cardiovascular health [1,2]. Current guidelines throughout the western world vary considerably; European countries recommend between 100 and 500 g of seafood per capita per week [3] while the FDA recommends around 225 g seafood per capita per week [4]. Specific guidelines are issued in relation to heart diseases; both the American Heart Association (AHA) [5] and the European Society of Cardiology (ESC) [6] recommend the consumption of two portions of fish per week (150 g/portion), which may provide up to 2 g of eicosapentanoic (EPA) and docosahexanoic (DHA) acids. EPA and DHA are considered the most important ω-3 fatty acids in seafood.

Inflammation has been linked to CVD [7], and various inflammation markers have been investigated in relation to fish consumption, but only few studies have shown a significant decrease in these markers [1]. C-reactive protein (CRP) was reduced only among subjects with high baseline CRP levels [8] or an increased body weight [9] while interleukins were mainly unaffected by fish consumption [1]. 

Fish oil consists mainly of unsaturated fatty acids, which are susceptible to oxidation; hence, many fish oil supplementation studies have measured oxidative stress markers. However, the findings are inconsistent, since ω-3 fatty acids seem to both promote and reduce oxidation [10,11]. Moreover, oxidized LDL (ox-LDL) [9,12] and glutathione peroxidase (GPx) [13,14] remain unaffected after fish consumption.

The platelet activating factor (PAF) is a bioactive lipid compound that is involved in a variety of pathophysiological conditions such as inflammation, atherosclerosis, and thrombosis. This phosphoglycerylether lipid is produced by most cell types either in basal conditions or after stimulation and is tightly linked with the mechanisms promoting CVD [15]. 

PAF levels are mainly regulated via enzymatic biosynthesis and catabolism. PAF biosynthesis follows two distinctive paths: the remodeling pathway, with the crucial enzyme being the calcium-dependent acetyl-CoA: lysoPAF acetyltransferase (lysoPAF-ATC), and the de novo pathway, with the enzyme dithiothreitol (DTT)-insensitive cytidine 5’-diphosphocholine (CDP-choline): 1-alkyl-2-acetyl-sn-glycerol cholinephosphotransferase (PAF-CPT) catalyzing the final step. PAF levels are downregulated intracellularly by a PAF specific acetylhydrolase (PAF-AH) that inactivates PAF into lyso-PAF and extracellularly by its plasma isoform lipoprotein-associated phospholipase-A_2_ (Lp-PLA_2_). The latter enzyme has been strongly related to LDL and many studies refer to high Lp-PLA_2_ as an independent biomarker for higher risk of CVD [16,17,18].

The existence of PAF inhibitors has been reported in typical foods of the Mediterranean dietary pattern, including fish [19,20,21,22]. Additionally, in a recent review that summarizes the in vivo effect of a Mediterranean type of diet on PAF metabolism and action, only one dietary intervention study with fish or fish oil consumption reduced PAF-induced platelet aggregation [23].

Furthermore, an increasing demand in fish and seafood generally has been recorded due to their beneficial effects on human health. This demand leads to the need to reduce the use of the already limited resources of fish oil and fish meal in fish feed by replacing them with plant-based oils. It has been demonstrated that the partial replacement of fish oil with olive pomace (OP) and olive pomace oil (OPO) in the diet of farmed gilthead sea bream results in fish lipids with higher in vitro inhibitory activity against PAF compared to gilthead sea bream fed with a typical fish oil (FO) diet [24]. It should be noted that OP and OPO polar lipid extracts contain PAF antagonists that are capable of inhibiting atherogenesis in hypercholesterolemic rabbits [25,26]. In general, bioactive polar lipids added in commonly consumed foods, such as fish, may benefit both health and the environment since these residues of the food industry, considered as waste, can be used in the production of functional foods.

The aim of the present study is to investigate whether the consumption of farmed gilthead sea bream fed with an olive pomace-enriched diet (EF) has a beneficial effect on inflammation, oxidative stress, and PAF enzyme activity compared to the consumption of the conventionally fed farmed gilthead sea bream (CF).

## 2. Materials and Methods

### 2.1. Study Design and Intervention

The design of the clinical trial was described in detail in a previous publication [27]. Briefly, it was a double-blind randomized crossover clinical trial, in which apparently healthy adults participated. The subjects were between 30 and 65 years old, with a body mass index (BMI) between 24.0 and 31.0 kg/m^2^, and were not frequent fish consumers (<150 g of cooked fish per week). Information about the compliance of the participants to the study protocol has been previously provided [27]. There were two periods of 8 weeks of fish consumption separated by a washout period of at least 4 weeks. Participants consumed either CF or EF twice per week and were equally distributed between the two treatments. More specifically, the fish species was gilthead seabream (*Sparus aurata* L.), and both types were provided by NIREUS Aquaculture S.A. (Koropi, Attica, Greece). The production method of fish feed enriched with olive processing byproducts is described in detail elsewhere [28]. 

### 2.2. Blood Collection, RBC and Leukocytes Separation

Blood was collected after 10 h of overnight fast to obtain serum, RBC, and leukocyte-rich plasma (LRP). BD Vacutainer^®^ Plus Plastic Serum Tubes (Becton, Dickinson and Co., Franklin Lakes, NJ, USA) were used for serum collection, in which blood was left to clot at room temperature (RT) for 1 h and was then centrifuged at 1500× *g* for 10 min at RT. The supernatant was aliquoted and stored at −80 °C. In order to collect RBCs, blood was collected in BD Vacutainer™ EDTA Plasma Tubes, and after appropriate washing RBCs were stored at −80 °C in the presence of BHT as previously described [29]. 

Blood was also collected in BD Vacutainer™ Heparin Plasma Tubes to separate the LRP. At first, blood with heparin was treated with a 3% *w*/*v* dextran (from Leuconostoc spp., Mr 450,000–650,000, Sigma, St. Louis, MO, USA) saline solution leading to a final concentration of 1% *w*/*v* dextran. The mixture was kept for 40 min at room temperature (RT) and then the leukocyte-rich supernatant was centrifuged at 500× *g* for 10 min at RT. The cell-rich pellet was treated with a lysis solution consisting of 155 mM NH_4_Cl, 10 mM KHCO_3_, and 0.1 mM EDTA for 5 min to lyse the contaminating erythrocytes. The whole mixture was centrifuged at 300× *g* for 10 min at RT, the supernatant was disposed, and the remaining pellet was resuspended in 1 mL of a buffer containing 50 mM Tris–HCl at pH 7.4. The leukocyte suspension was homogenized by sonication on ice (4 times × 10 s, 39% max power with intervals of 30 s). The leukocyte homogenate was aliquoted and stored at −80 °C.

### 2.3. Determination of Fatty Acid (FA) Composition in RBC

The analytical procedure has already been published [29]. Briefly, the RBC samples underwent a hemolysis and lipids were collected in the chloroform phase of a biphasic solution. The samples were esterified as fatty acid methyl esters (FAME) and the fatty acid profile was determined by gas chromatography (GC). The fatty acid content of the RBC membranes was expressed as a percentage (%) of total FA content.

### 2.4. Determination of Oxidation Stress Biomarkers

Oxidized LDL (ox-LDL) was measured in serum with an MDA-oxLDL ELISA kit (Biomedica Gruppe, Vienna, Austria). Resistance to oxidation in the presence of Cu^2+^ (lag time) and thiobarbituric acid-reactive substances (TBARS) were all measured in serum while GPx was measured in both serum and leukocytes. The experimental protocols have already been described in detail [30].

### 2.5. Determination of Inflammation Markers

Both hs-CRP and IL-6 were measured in serum using commercially available ELISA kits (hs-CRP ELISA KIT (DIAsource ImmunoAssays S.A, Louvain-la-Neuve, Belgium) and IL-6 ELISA KIT (Invitrogen, Waltham, MA, USA)).

### 2.6. Protein Determination

In the enzymatic assays that follow, the amount of LRP sample that was used was calculated by determining the protein concentration of the samples according to the Bradford method [31] at 595 nm using Coomassie Brilliant Blue G 250 (Sigma, St. Louis, MO, USA) as the coloring agent. Free fatty acid, low endotoxin bovine serum albumin (BSA) (Sigma, St. Louis, MO, USA) was used as the protein standard, the concentrations of the calibration curve being 0.125 to 2 μg BSA, and the samples were diluted 10-fold in distilled water.

### 2.7. Determination of PAF-AH and Lp-PLA_2_ Activity 

Both phospholipases’ activities were determined by the trichloroacetic acid (TCA) precipitation method using [^3^H] PAF as a substrate [32]. PAF-AH was determined in the leukocyte homogenates while Lp-PLA_2_ was determined in the serum. In brief, either LRP containing 60 μg of protein or 2 μL of serum were incubated with 4 nmol of [^3^H] PAF (20 Bq/nmol) for 15 min, at 37 °C, in a final volume of 200 μL of Tris-HCl 100 mM buffer (pH 7.2) containing 1 mM EGTA. The reaction was terminated by adding cold TCA (10% final concentration). After 30 min in an ice bath, samples were centrifuged at 15,000× *g* for 4 min at 4 °C. The [^3^H]-acetate released into the aqueous phase was measured on a liquid scintillation counter (Wallac Racbeta 1209, Pharmacia, Stockholm, Sweden). All assays were performed in duplicate. The enzyme activity was expressed as nmol PAF degraded/min/mg total protein (PAF-AH) or nmol PAF degraded/min/mL serum (LpPLA_2_).

### 2.8. Determination of DTT-Insensitive Cholinophosphotransferase Activity

DTT-insensitive CDP-choline:1-alkyl-2-acetyl-sn-glycerol cholinephosphotransferase (PAF-CPT) was determined in LRP. Cell homogenates, containing 20 μg protein, were incubated at 37 °C for 5 min with 11.25 mM DTT, 7.5 mM MgCl_2_, 0.375 mM EDTA, 1 mg/mL BSA, 0.1 mM CDP-choline, and 0.1 mM AAG (dissolved in DMSO) in a final volume of 200 μL of Tris-HCl 100 mM at pH 8. The reaction was stopped by adding cold chloroform:methanol 1:1 (2% acetic acid) following the Bligh–Dyer method of lipid extraction [33]. All assays were performed in duplicate. The chloroform phase containing the PAF was subjected to thin layer chromatography (TLC) to separate PAF from the lipid extracts. Glass plates were coated with Silica Gel G 60 (Merck) and the elution system that was used was chloroform:methanol:water:acetic acid 100:57:16:8. Lipid standards, containing sphingomyelin (Sm), lysophosphatidylcholine (LPC), phosphatidylcholine (PC), and phosphatidylethanolamine (PE), were used on each plate to identify the area of the plate containing PAF, between Sm and LPC, with the help of iodine vapors. The band containing PAF was scraped off and PAF was extracted using the Bligh–Dyer method. The chloroform phase containing the PAF was evaporated, diluted in 2.5 mg/mL BSA in saline, and quantified with the washed rabbit platelet aggregation assay (WRP) [34]. Platelet aggregation was measured at 37 °C in a 400-VS aggregometer coupled to a recorder, both provided by Chrono-Log (Havertown, PA, USA). A PAF standard in 2.5 mg/mL BSA in saline was used to construct the standard curve (5–20 fmol) that was used to determine the amount of PAF according to the aggregation that was induced by the samples. The enzymatic activity was expressed in pmol PAF/min/mg of total protein. The use of rabbits for experimental purposes was licensed by the Agricultural Economy & Veterinary department, Attica Region (3066/19 May 2014).

### 2.9. Determination of Calcium Dependent Lysopaf Acetyltransferase Activity

Calcium-dependent acetyl-CoA:lysoPAF acetyltransferase (lysoPAF-ATC) was also determined in LRP. Cell homogenates, containing 15 μg protein, were incubated at 37 °C for 10 min with 2 mM CaCl_2_, 0.2 mM acetyl-CoA, 20 μΜ lysoPAF, and 0.25 mg/mL BSA in a final volume of 200 μL of Tris-HCl 50 mM at pH 7.4. The reaction was stopped as mentioned in PAF-CPT assay above and the Bligh–Dyer method was used to obtain the lipids. All assays were performed in duplicate. The chloroform phase containing the PAF was subjected to TLC and PAF levels were determined by WRP as previously described in 2.8. 

### 2.10. Statistical Methods

All statistical analyses were performed using Stata Statistical Software, Release 12 (StataCorp LP: College Station, TX, USA). The significance (α) level was set to 0.05 for all tests. The Shapiro–Wilk test and normal Q–Q plots were used to test normal distribution of data. Data were analyzed based on the per-protocol principle.

The descriptive statistics summarizing data about the demographic, anthropometric, standard biochemical, physical activity-related, and dietary characteristics of the participants in the two sequence groups (CF/EF vs. EF/CF) assessed at the beginning of the study have been previously published [27]. Data about markers of inflammation and oxidative stress, the enzymes of PAF metabolism, and the fatty acid composition of the RBC membranes for the two sequence groups assessed at the beginning of the study are summarized in the present paper; the results are presented as median (25th and 75th percentile). Mann–Whitney tests were used to compare CF/EF versus EF/CF group, and *p*-values are presented for two-sided tests.

Endpoints for the RBC membrane fatty acid composition (%), markers of inflammation and oxidative stress, and the enzymes of PAF metabolism are presented separately for each fish and period of treatment; data are summarized as median (25th and 75th percentile). Input data are also expressed as relative change (%), calculated as the change between values at the end and values at the beginning of each treatment period. Wilcoxon matched-pairs signed-rank tests were used to compare the values at the end of each treatment with the values at the beginning of the same treatment for the combined results from the two periods, and *p*-values are presented for two-sided tests.

Comparisons between the EF and the CF following 8 weeks of intervention, and estimations of the effect size, were performed for the enzymes of PAF metabolism with multilevel mixed-effects linear regression. First, the treatment effect was estimated with the fish type as the exposure and the outcome variable evaluated at the end of the intervention period as the dependent variable; adjustments were made for the sex of participants (men versus women), the affiliation to sequence groups (EF/CF versus CF/EF), and the outcome variable evaluated at the beginning of each period. Second, the effect of the order of the received treatment was also evaluated with the interaction factor “Treatment × Sequence” as the exposure and the same dependent variable as above; the outcome variable evaluated at the beginning of each period, as well as the sex of the participants, were entered as covariates. In the context of the mixed-effects linear regression, random effects were also specified at the level identified by the variable ID, that is, the participant level. Residuals from the models were graphically checked for normal distribution (histograms) and homoscedasticity (plot of the residuals versus fitted values); dependent variables were ln-transformed due to departures from normal distribution and homoscedasticity of the residuals. Results are presented as mean effect (standard error, SE), 95% confidence interval (95% CI) of the effect, and *p*-values; the percent changes, calculated as the exponentiated value of the coefficient for the comparison of EF against CF (reference group), are also reported. Similar analyses were performed for the markers of inflammation and oxidative stress, as well as the fatty acid composition of the RBC membranes; however, only statistically significant results are reported. Regarding the effect of fish consumption on the fatty acid profile of the RBC membranes, 18 fatty acids were individually evaluated with mixed-effects linear regression. Since fatty acids were measured on the same gas chromatography platform, we adjusted the *p*-values that correspond to multiple testing when examining the effect of treatment on each fatty acid contained in the RBC membrane lipids. Adjustments were performed with the *qqvalue* package in Stata using the Benjamini–Hochberg multiple-test procedure, and *q*-values (adjusted *p*-values) were calculated. Estimated mean effects were considered significant when *q* < 0.05.

Associations between the activity of each enzyme of PAF metabolism (i.e., PAF-CPT, Lyso-PAF AT, PAF-AH, and LpPLA_2_) and the % fatty acid content of the RBC membranes—for the fatty acids selected based on the fish treatments administered in the present study—were calculated using Spearman’s rank correlation. Input data were fold changes, i.e., the ratio of values at the end to values at the beginning of each treatment period. Analyses were performed by “Sequence”, “Treatment”, and “Treatment × Sequence”. Only statistically significant associations are reported.

## 3. Results

The flow diagram of the study is provided in the Supplementary Material (Figure S1).

### 3.1. Characteristics of Sequence Groups at the Beginning of the Study

The anthropometric and biochemical characteristics of the participants at the beginning of the study were similar between the two groups [27]. In addition, no significant differences were found for the inflammation markers (i.e., hs-CRP and IL-6), the oxidative stress markers (i.e., TBARS, ox-LDL, resistance of serum to oxidation, GPx in serum, and GPx in leukocytes), or the enzymes of PAF metabolism (i.e., LpPLA_2_, Lyso-PAF ATC, PAF-AH, PAF-CPT, and the LpPLA_2_-to-LDL ratio) between the CF/EF and the EF/CF groups at the beginning of the study (Table 1). For both inflammation markers, hs-CRP and IL-6, the levels were within the ranges provided by the ELISA kits that were used (0.1–10 μg/mL for hs-CRP and 2–1000 pg/mL for IL-6), although they were slightly elevated compared to the respective 50^th^ percentile, implying the existence of a low-grade inflammation possibly due to the participants being overweight. Furthermore, the two groups did not differ with respect to the fatty acid composition (%) of the red blood cells (RBC), except for the saturated fatty acid 22:0 and the ω-3 polyunsaturated fatty acid 22:6 (Table S1).

### 3.2. Endpoints and Changes in Outcomes after Fish Treatments

Table 2 and Table 3 summarize the data on the markers of inflammation and oxidative stress, as well as the activity of the enzymes of PAF metabolism at the end of the first and second periods of consumption of the CF or EF, respectively. High-sensitivity CRP, ox-LDL, the lag time to serum oxidation, and the PAF-CPT activity were significantly decreased at the end of the CF intervention (*p* values are shown in Table 2 for the combined results from the two periods). The LpPLA_2_-to-LDL ratio was significantly decreased at the end of the EF intervention (*p* values are shown in Table 3 for the combined results from the two periods). It should be noted that EF had an opposite effect on PAF-CPT activity at the end of each period, and overall, no significant effect was observed, whereas CF significantly reduced PAF-CPT activity in the second period. After EF consumption, the ox-LDL decreased and the GPx in serum increased, both of which were borderline significant. Similarly, Tables S2 and S3 summarize data with respect to the fatty acid composition of the RBC membranes at the end of the first and second periods of consumption of the CF or the EF, respectively. In addition, the percent changes in the outcomes indicate that the values at the beginning may have a significant impact on the values at the end of each treatment period; thus, the variables evaluated at the beginning of each treatment period are used as covariates in the subsequent analyses.

### 3.3. Effect of Fish Type Consumed on the Enzymes of PAF Metabolism

Following 8 weeks of treatment, no statistically significant changes were found for LpPLA_2_, the LpPLA_2_-to-LDL ratio, PAF-AH, or Lyso-PAF ATC when the EF was compared to the CF (Table 4). In contrast, a statistically significant increase was found for the PAF-CPT activity (the arithmetic mean of the ln-transformed variable) after 8 weeks of treatment with the EF compared to the CF (coefficient = 0.194; *p* = 0.033; Table 4); consequently, this translates to a 21% increase in PAF-CPT activity when switching from the CF to the EF. 

Statistically significant order effects were found for PAF-AH and PAF-CPT (Table S4). With respect to PAF-AH, a statistically significant decrease was found when the EF was compared to the CF in the CF/EF sequence only; the mean decrease in the ln-transformed PAF-AH activity was −0.328 (*p* = 0.019), which is a 28% decrease in the PAF-AH activity following 8 weeks of treatment with the EF compared to the CF in the CF/EF sequence. In contrast, a statistically significant increase was found for PAF-CPT when the EF was compared to the CF in the EF/CF sequence; the mean increase in the ln-transformed PAF-CPT activity was 0.367 (*p* = 0.004) or 44% in the PAF-CPT activity following 8 weeks of EF treatment compared to the CF in the EF/CF sequence. Similarly, a 33% increase in PAF-CPT activity was found (*p* = 0.027) following 8 weeks of EF treatment in the EF/CF sequence compared to the CF treatment in the CF/EF sequence.

### 3.4. Effect of Fish Type Consumed on Markers of Inflammation and Oxidative Stress

Both fish types were similar as no statistically significant treatment effects were found for any of the inflammation markers (hs-CRP and IL-6) or the oxidative stress markers (TBARS, ox-LDL, lag time (resistance of serum to oxidation), GPx in serum and GPx in leukocytes) when the EF was compared to the CF. Accordingly, no significant order effects were observed. 

### 3.5. Effect of Fish Type Consumed on the Fatty Acid Composition of the RBC Membranes

The intake of both fish types resulted in the elevation of the EPA and DHA content of the RBC membranes, as previously reported [27]. In addition, the CF consumption resulted in the higher arachidic acid content (20:0) of the RBC membranes (Table S2) while the EF consumption increased the lignoceric acid (24:0) and nervonic acid (24:1 ω-9) content and decreased the oleic acid content(18:1 ω-9) (Table S3).

A statistically significant treatment effect was found for the fatty acid composition (%) of the RBC membranes regarding the palmitic acid (16:0) and the arachidonic acid (AA, 20:4 ω-6) (*p* = 0.024 and 0.003, respectively, Table 5). After adjusting for the false-positive discovery rate (FDR), the mean content (%) of AA in the RBC membrane lipids tended to be significantly lower when the EF treatment was compared to the CF treatment (*q* = 0.054, Table 5), whereas no significant difference was found for the mean content (%) of palmitic acid (*q* = 0.216, Table 5).

Statistically significant order effects were found for the palmitic acid (16:0) and the AA (20:4 ω-6) (*p* = 0.042 and 0.003, respectively). In particular, a statistically significant increase in the palmitic acid content (%) of the RBC membrane lipids was found following 8 weeks of treatment with the EF in the EF/CF sequence compared to the CF in the CF/EF sequence (mean effect (SE): 1.39 (0.69); 95% CI: 0.05, 2.74). In contrast, a statistically significant decrease in the AA content (%) of the RBC membrane lipids was found when the EF was compared to the CF in the CF/EF sequence only (mean effect (SE): −0.73 (0.24); 95% CI: −1.21, −0.25).

### 3.6. Correlations between RBC Membrane Fatty Acids and PAF Metabolic Enzymes

In the CF treatment, a significant negative correlation was found between PAF-CPT activity and EPA (*p* = 0.002), DHA (*p* = 0.025), and the sum of the ω-3 fatty acids (*p* = 0.018). In the EF treatment, a significant negative correlation was found between PAF-AH activity and EPA (*p* = 0.008), DHA (*p* = 0.007) and the sum of the ω-3 fatty acids (*p* = 0.028).

When both the type of fish and the sequence of the interventions are considered, the CF treatment in the CF/EF sequence exhibits a negative correlation between PAF-CPT activity and EPA (*p* = 0.008) and a positive correlation between PAF-CPT activity and AA (*p* = 0.025) as well as the sum of the ω-6 fatty acids (*p* = 0.013). The CF treatment in the EF/CF sequence exhibits a negative correlation between LpPLA_2_ and EPA (*p* = 0.048). Regarding the EF treatment in the CF/EF sequence, a negative correlation is found between PAF-AH activity and DHA (*p* = 0.042), as well as the sum of ω-3 fatty acids (*p* = 0.042), whereas a positive correlation is found between LysoPAF-ATC activity and AA (*p* = 0.019) in the EF treatment in the EF/CF sequence.

## 4. Discussion

Fish consumption has long been considered a beneficial dietary habit regarding the prevention of cardiovascular disease. The positive effects of fish consumption are mostly attributed to the ω-3 fatty acids existing in fish species. Moreover, fish contains bioactive polar lipids rich in ω-3 PUFA with antithrombotic and anti-inflammatory properties [2]. Therefore, it seems reasonable to investigate the possible effect of fish consumption on potent inflammatory lipid mediators such as PAF. In addition, the consumption of the EF may lead to an improved inflammation profile compared with CF. 

In addition, over the past few years, lipid sources originating from plants have been studied in an attempt to reduce the dependence of the aquaculture industry on fish oil sources so as to achieve the sustainable production of fish feeds. Olive pomace (OP), a major by-product of the olive oil extraction industry, has arisen as a promising alternative lipid source of plant origin, partly due to its competitive price in comparison to other plant-based lipid sources. In addition, fish feed formulation requires only a small amount of OP as opposed to other plant-based lipid sources [35]. In the trial presented herein, olive pomace was substituted for 8% of the fish oil in the diet of farmed gilthead sea bream (*Sparus aurata* L.) without compromising growth performance of this fish species [24].

The previously published results from in vitro experiments on the total lipids (TL) and total polar lipids (TPL) obtained from the fillets of gilthead sea bream fed with the 100% fish oil (FO) diet and from the fillets of fish fed with the OP-enriched diet indicated that OP enhances the inhibitory activity of gilthead sea bream against PAF-induced platelet aggregation [24,36]. Despite the promising findings from the in vitro studies, we did not previously observe any greater effect on platelet aggregation, especially PAF-induced aggregation, after the consumption of gilthead sea bream fed with the OP-enriched diet compared to fish fed with the FO diet among apparently healthy adults [27]. Instead of targeting the interaction of PAF with its receptor in order to prevent the initiation of complex pathways that are in the junction of coagulation and inflammation cascades, future research may also focus on altering PAF metabolism as a means to beneficially modulate PAF levels [37].

In the present study, the activities of PAF-CPT, lysoPAF-AT, PAF-AH, and LpPLA_2_ were measured before and after EF or CF consumption. The baseline levels of all the enzymes were similar to those of previous studies where the same experimental protocols were used [38,39,40,41]. LysoPAF-AT was neither affected by any treatment nor presented significant changes when the two interventions were compared. Regarding PAF-CPT, its activity was significantly decreased after the CF intake and even though EF on its own had no significant effect, its intake increased PAF-CPT activity by 20% compared to CF. This result contrasts with the decrease of PAF-CPT activity after the consumption of a similarly enriched yogurt in a recent study [40]. In the same study, the participants who consumed the enriched yogurt had lower activities of LpPLA_2_ in serum and PAF-AH in leucocyte homogenate compared to participants who consumed the plain yogurt at the end of the treatment, although these differences were not statistically significant. The findings from the present study comparing EF to CF showed that the activities of PAF-AH, LpPLA_2_, and the LpPLA_2_-to-LDL ratio were not significantly different between the two types of fish. However, EF consumption resulted in a significantly lower LpPLA_2_-to-LDL ratio and the order effect analyses showed that the PAF-AH activity was significantly decreased after the EF compared to the CF consumption in the CF/EF sequence.

Nevertheless, the beneficial effects of intake of both types of fish can be seen apart from the elevation of the EPA and DHA concentration in the RBC membranes, since CF consumption also increased arachidic acid (20:0) and EF consumption increased lignoceric acid (24:0). These very long-chain saturated fatty acids have been associated with a lower risk of heart failure [42]. The consumption of the EF also increased 24:1 ω-9 and decreased 18:1 ω-9 while gondoic acid (20:1 ω-9) remained unaffected in the RBC membranes, implying a shift of monounsaturated fatty acid metabolism from 18:1 ω-9 to 24:1 ω-9. More importantly, the EF intake resulted in the reduction of AA concentration in the RBC membranes, a lipid that is considered as a key inflammatory intermediate and is a precursor to a wide range of eicosanoids. In addition, AA is the predominant fatty acid at the sn-2 position of ether-linked phospholipids such as PAF and it is well established that it is an essential substrate in the remodeling pathway of PAF biosynthesis [43]. In the EF group, a negative correlation between the AA and PAF-CPT activity was recorded as well as a positive correlation of AA with lysoPAF-AT in the same intervention group. These findings may explain the increase of the PAF-CPT activity since the decrease of AA could lead to the reduction of PAF production by the remodeling pathway and subsequently to the enhanced production of PAF through the de novo biosynthesis by the activation of PAF-CPT. Fragopoulou et al. also reported that a dietary pattern low in ω-6 fatty acids was inversely related with PAF-CPT in healthy subjects [29]. Furthermore, the negative correlation between ω-3 fatty acids EPA and DHA as well as PAF-CPT only in the CF group may suggest that the ω-3 fatty acids consumed during the clinical trial were borderline sufficient for provoking alternations in the enzymatic activities. On that note, there are also negative correlations of ω-3 fatty acids with PAF-AH and its cytosolic isoform LpPLA_2_, yet they are sparse among the groups. These results could explain that fish consumption, although reasonable in terms of a usual diet, is not capable of provoking significant changes. Previous studies regarding the association of RBC fatty acids and PAF enzymes in healthy subjects did not observe any relation between ω-3 fatty acids and the enzymes [29,44].

It has also been suggested that atherosclerosis is strongly related to inflammation and oxidative stress. A series of inflammation and oxidation markers were measured in the present study to assess any change that may occur after a fish intervention. The baseline levels were similar to other studies [30,45] and any small differences may be attributed to different protocols. Comparing EF and CF consumption did not lead to any significant differences regarding those markers. However, there were some significant results when the data were grouped for type of fish and period of intervention. Ox-LDL showed a reduction in both EF and CF, and hs-CRP was also lower after CF consumption. This result partially agrees with other clinical trials on fish consumption [1] and may be attributed to the fact that the participating population was in good health; hence, any beneficial effects of fish consumption towards inflammation and/or oxidative stress might have been too subtle to be observed.

The results of the present study suggest that there may be a subtle effect on PAF enzymes after frequent fish consumption. It has not been clarified whether the EF has significant differences from the CF, as far as their effect on PAF metabolism is concerned. Considering that the participants in the present study were rather healthy, it is possible that two fish meals per week may not be enough to provoke significant alterations on PAF enzymes as well as inflammation and oxidative stress markers. Similar protocols with more meals per week, that include subjects with established cardiovascular or inflammatory conditions, or that perform the enrichment of the fish feed with the isolated olive pomace polar lipid fraction, containing the bioactive specific PAF-receptor antagonists, may be more suitable for observing the effect of potential PAF inhibitors such as fish lipids and micro-constituents as well as the effects of bioactive lipids from olive pomace.

### Limitations and Strengths

The eligible subjects were apparently healthy overweight adults of Greek ethnicity, so caution is needed in the extrapolation of the findings to different populations, e.g., younger or older, of normal body mass index or obese, suffering from a medical disorder, or belonging to other ethnic groups. The sample size was calculated based on the PAF concentration required to induce 50% platelet aggregation (EC_50_); therefore, the study may be underpowered with respect to the outcomes evaluated in the present paper. We did not measure the blood levels of PAF, a piece of information that could be interpreted along with the activity of the evaluated PAF enzymes. Finally, only participants with non-missing data were included in the analyses, which introduced a bias in the parameter estimation.

The crossover design of the study is an important strength since each participant acts as their own control and the variability between participants is eliminated. The compliance of participants to the study protocol was evaluated with subjective and objective tools and methods, i.e., self-administered records, phone calls, and the fatty acid analysis of the red blood cell membrane lipids. Special care was taken regarding the treatment allocation and data assessment. The absence of differences between the two sequence groups at the beginning of the study with respect to the outcomes of interest, i.e., the activities of the enzymes of PAF metabolism, adds to the value of the findings. To our knowledge, this is the first study that has investigated the impact of two differently fed fish—one being the product of the long-standing practice in the aquaculture industry and the other being a novel product of sustainable fish farming—on PAF metabolism among humans.

## 5. Conclusions

In conclusion, this is the first study to evaluate the impact of differently fed gilthead sea bream consumed twice weekly on the activity of key enzymes of PAF metabolism, i.e., PAF-CPT, Lyso-PAF AT, PAF-AH, and LpPLA_2_, among apparently healthy overweight adults. The consumption of fillets from gilthead sea bream fed with a diet where fish oil in the grow-out fish feed was partly replaced with olive pomace did not have a greater impact on Lyso-PAF AT, the regulating enzyme of the remodeling PAF biosynthesis pathway, or PAF-AH and LpPLA_2_, the intracellular and extracellular isoforms of the PAF catabolic pathway, compared to the consumption of fillets from fish fed the typical fish-oil diet. The increased PAF-CPT activity after EF consumption can be attributed to regulating mechanisms following the AA reduction observed in the same intervention group.

## Figures and Tables

**Table 1:** Inflammation markers, oxidative stress markers, and enzymes of PAF metabolism of participants at the beginning of the study by sequence.

	CF/EF	EF/CF	*p*
Participants, n (%)	15 (50)	15 (50)	
Sex			0.272
Male, n (%)	10 (33.3)	6 (20.0)	
Age (years)	42 ± 6	45 ± 8	0.360
**Inflammation markers**			
hs-CRP (μg/mL)	3.47 (1.62, 8.57)	3.66 (1.60, 10.06)	1.000
IL-6 (pg/mL)	29 (18, 75)	32 (20, 168)	0.678
**Oxidative stress markers**			
TBARS (μM)	1.60 (1.29, 2.04)	1.70 (1.33, 5.39)	0.575
ox-LDL (μg/mL)	0.88 (0.47, 3.17)	1.43 (0.57, 2.86)	0.507
Lag time (min)	58.4 (54.0, 71.0)	55.2 (49.0, 93.3)	0.820
GPx in serum (U/mL)	0.064 (0.059, 0.077)	0.060 (0.052, 0.071)	0.213
GPx in leukocytes (U/min/mg)	0.050 (0.033, 0.073)	0.044 (0.036, 0.058)	0.756
**PAF enzymes**			
LpPLA_2_ (nmol/min/mL)	17.33 (13.04, 22.37)	17.43 (12.40, 19.57)	0.663
LpPLA_2_-to-LDL ratio	0.13 (0.11, 0.16)	0.13 (0.08, 0.17)	0.576
PAF-AH (pmol/min/mg)	84.55 (66.39, 121.86)	77.05 (51.30, 121.12)	0.633
PAF-CPT (pmol/min/mg)	612.9 (404.3, 840.6)	459.6 (375.4, 631.6)	0.330
Lyso-PAF ATC (pmol/min/mg)	3.34 (2.85, 6.25)	5.56 (2.66, 10.24)	0.663

Categorical variables are summarized as frequencies, n (%); continuous variables are summarized as median (25th and 75th percentile), except for age, which is summarized as mean ± standard deviation. Mann–Whitney tests were used to compare sequence groups for continuous variables with non-normal distribution; *p*-values are presented for two-sided tests. Significance (α) level is set to 0.05. hs-CRP: high sensitivity C-reactive protein; GPx: glutathione peroxidase; IL-6: interleukin 6; lag time (resistance of serum to oxidation); LpPLA_2_: lipoprotein-associated phospholipase A_2_; ox-LDL: oxidized low-density lipoprotein; Lyso-PAF ATC: calcium-dependent lyso-platelet-activating factor acetyltransferase; PAF-AH: platelet-activating factor acetyl-hydrolase; PAF-CPT: platelet-activating factor-cholinephosphotransferase. TBARS: thiobarbituric acid-reactive substances.

**Table 2:** Inflammation markers, oxidative stress markers, and enzymes of PAF metabolism of the participants on the conventional fish (CF) diet by period.

	End of 1st Period	% Change	End of 2nd Period	% Change	*p* *
**Inflammation** **markers**					
hs-CRP (μg/mL)	2.33 (1.16, 5.62)	−33.89 (−58.16, −4.10)	2.46 (0.5, 5.19)	−21.36 (−77.82, 1.36)	**0.006**
IL-6 (pg/mL)	29 (19, 72)	−5.0 (−11.11, 5.56)	32 (18, 135)	−5.56 (−25.0, 6.25)	0.087
**Oxidative stress markers**					
TBARS (μM)	1.73 (1.37, 2.02)	−0.98 (−9.74, 7.69)	1.92 (1.6, 6.07)	11.93 (2.56, 22.58)	0.082
ox-LDL (μg/mL)	0.81 (0.49, 2.69)	−7.02 (−31.97, 1.59)	1.61 (0.59, 2.67)	−8.77 (−15.79, −3.40)	**0.006**
Lag time (min)	60.6 (46.3, 69.6)	−3.42 (−15.15, 11.28)	56.9 (46.4, 62.3)	−10.25 (−19.39, 3.04)	**0.037**
GPx in serum (U/mL)	0.073 (0.067, 0.078)	10.50 (−5.10, 24.20)	0.065 (0.057, 0.075)	−0.95 (−12.56, 12.29)	0.329
GPx in leukocytes (U/min/mg)	0.048 (0.033, 0.062)	−5.47 (−14.72, 30.08)	0.057 (0.040, 0.069)	12.93 (−19.01, 49.95)	0.199
**PAF enzymes**					
LpPLA_2_ (nmol/min/mL)	19.15 (13.09, 20.13)	−2.51 (−15.17, 4.59)	17.72 (11.82, 21.26)	−3.11 (−8.73, 11.17)	0.178
LpPLA_2_-to-LDL ratio	0.12 (0.09, 0.14)	−10.92 (−26.12, −0.20)	0.15 (0.07, 0.21)	4.43 (−8.02, 14.37)	0.111
PAF-AH (pmol/min/mg)	97.57 (77.09, 156.02)	11.17 (−20.20, 64.34)	64.56 (57.51, 100.63)	6.53 (−28.11, 37.47)	0.453
PAF-CPT (pmol/min/mg)	466.9 (305.0, 722.9)	−1.0 (−30.78, 15.48)	534.9 (319.0, 678.7)	−18.18 (−34.66, −3.04)	**0.019**
Lyso-PAF ATC (pmol/min/mg)	4.48 (3.0, 6.45)	9.94 (−5.99, 55.04)	5.28 (3.55, 11.71)	0.38 (−3.99, 86.80)	0.125

Data are summarized as median (25th and 75th percentile). Data are also expressed as relative change (%), calculated as the change between values at the end and values at the beginning of each treatment period. * Wilcoxon matched-pairs signed-rank tests were used to compare the values at the end of treatment with the values at the beginning of treatment for the combined results from the two periods; *p*-values are presented for two-sided tests; the significance (α) level was set to 0.05 for all two-sided tests.

**Table 3:** Inflammation markers, oxidative stress markers, and enzymes of PAF metabolism of the participants on the enriched fish (EF) diet by period.

	End of 1st Period	% Change	End of 2nd Period	% Change	*p* *
**Inflammation** **markers**					
hs-CRP (μg/mL)	4.51 (0.43, 8.91)	−12.20 (−73.13, 38.89)	3.51 (1.56, 5.0)	−19.78 (−45.87, 79.93)	0.673
IL-6 (pg/mL)	41 (18, 152)	0.0 (−10.17, 68.0)	33 (17, 72)	−5.56 (−16.67, 1.77)	0.665
**Oxidative stress markers**					
TBARS (μM)	1.85 (1.27, 2.36)	−2.23 (−15.27, 6.72)	1.69 (1.35, 2.33)	1.81 (−7.48, 10.39)	0.992
ox-LDL (μg/mL)	1.78 (0.55, 2.7)	−6.67 (−12.18, 17.05)	0.62 (0.45, 1.88)	−4.94 (−14.16, 5.56)	0.067
Lag time (min)	61.3 (49.3, 81.4)	−1.56 (−12.39, 9.17)	58.7 (51.5, 72.4)	−2.28 (−6.57, 6.73)	0.417
GPx in serum (U/mL)	0.066 (0.061, 0.070)	7.0 (0.26, 13.54)	0.071 (0.067, 0.078)	2.92 (−6.57, 12.86)	0.057
GPx in leukocytes (U/min/mg)	0.047 (0.039, 0.052)	6.10 (−10.02, 30.29)	0.057 (0.044, 0.062)	21.74 (−9.66, 35.91)	0.090
**PAF enzymes**					
LpPLA_2_ (nmol/min/mL)	15.70 (10.36, 17.18)	−1.57 (−14.78, 3.26)	16.26 (10.45, 22.04)	−7.19 (−15.73, 4.0)	0.050
LpPLA_2_-to-LDL ratio	0.09 (0.07, 0.15)	−15.52 (−31.22, −4.85)	0.13 (0.07, 0.20)	−6.57 (−13.51, 2.34)	**0.001**
PAF-AH (pmol/min/mg)	86.46 (56.09, 112.0)	12.72 (−12.66, 35.78)	70.72 (57.16, 145.03)	−9.21 (−21.22, 29.10)	0.829
PAF-CPT (pmol/min/mg)	557.0 (412.4, 790.5)	10.72 (−1.98, 69.04)	544.3 (483.3, 659.5)	−13.64 (−24.20, 3.03)	0.877
Lyso-PAF ATC (pmol/min/mg)	6.88 (3.15, 12.01)	21.68 (−8.40, 121.83)	4.46 (3.01, 7.63)	13.66 (−22.09, 34.55)	0.106

Data are summarized as median (25th and 75th percentile). Data are also expressed as relative change (%), calculated as the change between values at the end and values at the beginning of each treatment period. * Wilcoxon matched-pairs signed-rank tests were used to compare the values at the end of treatment with the values at the beginning of treatment for the combined results from the two periods; *p*-values are presented for two-sided tests; the significance (α) level was set to 0.05 for all two-sided tests.

**Table 4:** Effect of the enriched fish (EF) versus the conventional fish (CF) diet on the activity of PAF metabolic enzymes (treatment effect).

	Mean Effect (SE)	95% CI	*p*
LpPLA_2_	−0.043 (0.039)	−0.119, 0.033	0.269
LpPLA_2_-to-LDL ratio	−0.044 (0.055)	−0.151, 0.063	0.424
PAF-AH	−0.113 (0.105)	−0.319, 0.092	0.279
PAF-CPT	0.194 (0.091)	0.016, 0.371	**0.033**
Lyso-PAF ATC	0.021 (0.072)	−0.119, 0.162	0.766

Dependent variables are ln-transformed. Treatment with fish is the exposure, and the activity of PAF metabolic enzymes evaluated at the end of the intervention period is the dependent variable; adjustments were made for the sex of participants (men versus women), the affiliation to sequence groups (EF/CF versus CF/EF), and the activity of PAF metabolic enzymes evaluated at the beginning of each period. Results are presented as mean effect (standard error, SE), 95% confidence interval (95% CI) of the effect, and *p*-values are also reported.

**Table 5:** Effect of the enriched fish (EF) versus the conventional fish (CF) diet on red blood cell (RBC) membrane fatty acid composition (%) (treatment effect).

	Mean Effect (SE)	95% CI	*p*	*q*
**SFA**				
Myristic acid (14:0)	0.019 (0.049)	−0.077, 0.115	0.700	0.841
Palmitic acid (16:0)	0.469 (0.208)	0.061, 0.877	**0.024**	0.216
Margaric acid (17:0)	−0.013 (0.019)	−0.051, 0.024	0.489	0.734
Stearic acid (18:0)	−0.046 (0.171)	−0.381, 0.290	0.789	0.841
Arachidic acid (20:0)	−0.167 (0.128)	−0.417, 0.084	0.192	0.576
Heneicosylic acid (21:0)	0.086 (0.100)	−0.111, 0.283	0.391	0.691
Behenic acid (22:0)	0.013 (0.051)	−0.087, 0.114	0.794	0.841
Lignoceric acid (24:0)	0.120 (0.078)	−0.032, 0.273	0.122	0.549
**MUFA**				
Vaccenic acid (18:1 ω-7)	0.010 (0.029)	−0.047, 0.067	0.728	0.841
Oleic acid (18:1 ω-9)	−0.215 (0.162)	−0.533, 0.104	0.186	0.576
Gondoic acid (20:1 ω-9)	−0.073 (0.069)	−0.209, 0.063	0.295	0.664
Erucic acid (22:1 ω-9)	0.071 (0.089)	−0.102, 0.245	0.422	0.691
Nervonic acid (24:1 ω-9)	0.135 (0.074)	−0.010, 0.281	0.068	0.408
**PUFA**				
Linoleic acid (18:2 ω-6)	0.126 (0.152)	−0.171, 0.423	0.406	0.691
Arachidonic acid (20:4 ω-6)	−0.521 (0.173)	−0.861, −0.182	**0.003**	**0.054**
Eicosapentaenoic acid (20:5 ω-3)	0.010 (0.083)	−0.154, 0.173	0.909	0.909
Docosapentaenoic acid (22:5 ω-3)	0.025 (0.047)	−0.068, 0.118	0.605	0.838
Docosahexaenoic acid (22:6 ω-3)	0.149 (0.137)	−0.122, 0.414	0.287	0.664

Treatment with fish is the exposure, and the fatty acid composition (%) evaluated at the end of the intervention period is the dependent variable; adjustments were made for the sex of participants (men versus women), the affiliation to sequence groups (EF/CF versus CF/EF), and the fatty acid composition (%) evaluated at the beginning of each period Results are presented as mean effect (standard error, SE), 95% confidence interval (95% CI) of the effect; *p*-values and *q*-values (adjusted *p*-values) are also reported. Arachidic acid and eicosapentaenoic acid are ln-transformed. MUFA: mono-unsaturated fatty acid; PUFA: polyunsaturated fatty acid; SFA: saturated fatty acid.

## Data Availability

The data of the present study are available from the corresponding author upon request.

## Supplementary Materials

The following supporting information can be downloaded at: https://www.mdpi.com/article/10.3390/foods11142105/s1, Figure S1: CONSORT flow diagram for crossover trials. CF: conventional fish; EF: enriched fish; Table S1: Red blood cell (RBC) membrane fatty acid composition (%) of participants at the beginning of the study by sequence; Table S2: Red blood cell (RBC) membrane fatty acid composition (%) of the participants on the conventional fish (CF) by period; Table S3. Red blood cell (RBC) membrane fatty acid composition (%) of the participants on the enriched fish (EF) by period; Table S4. Effect of the enriched fish (EF) versus the conventional fish (CF) on the activity of PAF metabolic enzymes depending on sequence (order effect).
