# Supplementary material for: Consumption of Farmed Fish, Fed with an Olive-Pomace Enriched Diet, and Its Effect on the Inflammatory, Redox, and Platelet-Activating Factor Enzyme Profile of Apparently Healthy Adults: A Double-Blind Randomized Crossover Trial
Source: Foods. 2022 Jul 15;11(14):2105. doi: 10.3390/foods11142105 (PMC9318848; doi:10.3390/foods11142105)
Supplement: Supplementary file 1 [file foods-11-02105-s001.zip › foods-1800658-supplementary.pdf]

Figure S1. CONSORT flow diagram for crossover trials. CF: conventional fish; EF: enriched fish

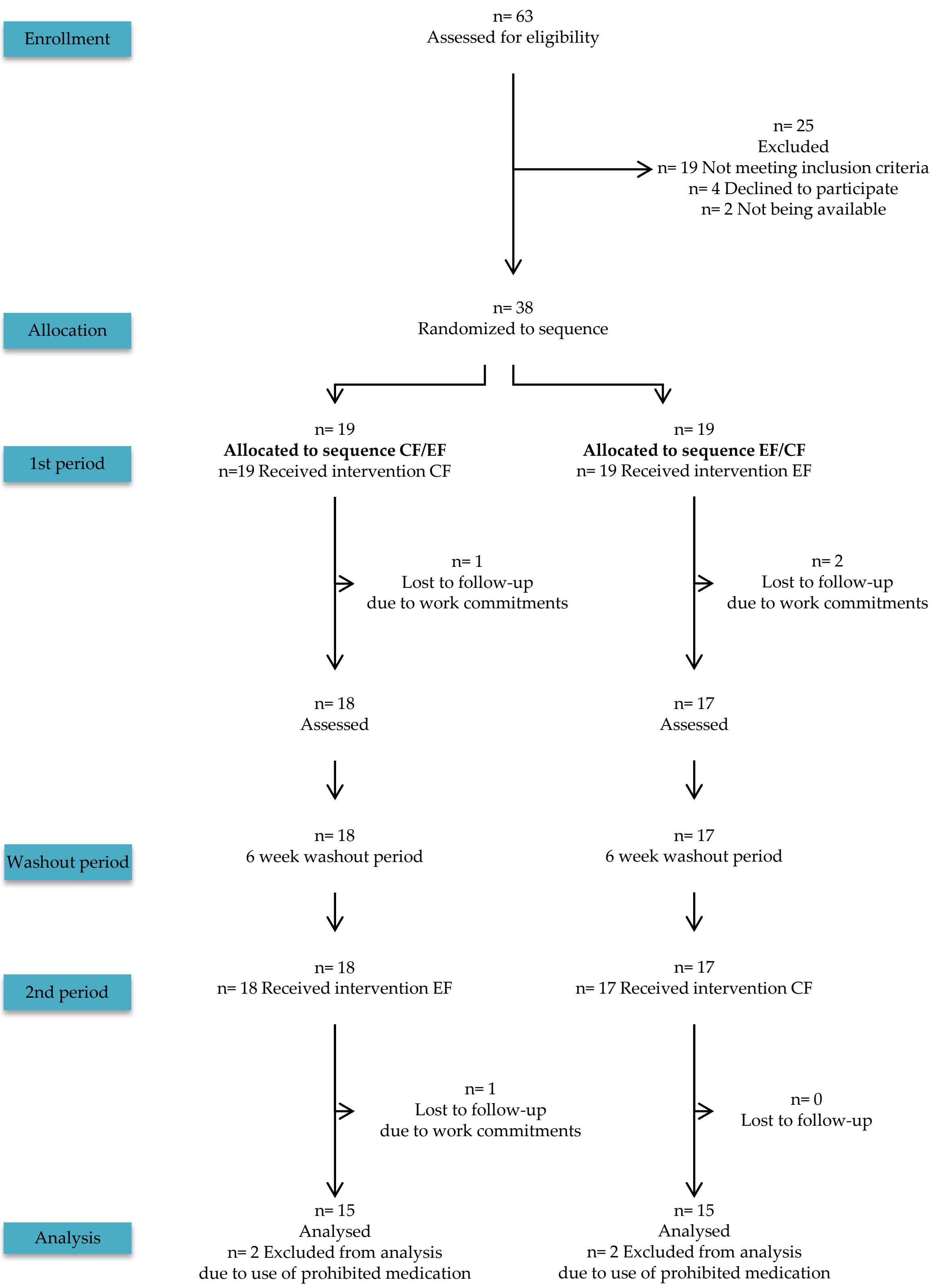

Table S1. Red blood cell (RBC) membrane fatty acid composition (%) of participants at the beginning of the study by sequence.

	**CF/EF**	**EF/CF**	***p***
SFA			
Myristic acid (14:0)	0.30 (0.23, 0.47)	0.35 (0.24, 0.42)	0.860
Palmitic acid (16:0)	22.20 (20.92, 22.99)	23.99 (20.73, 24.61)	0.157
Margaric acid (17:0)	0.48 (0.43, 0.53)	0.47 (0.37, 0.53)	0.626
Stearic acid (18:0)	16.50 (16.30, 17.16)	16.77 (16.30, 17.45)	0.752
Arachidic acid (20:0)	0.48 (0.39, 0.52)	0.45 (0.35, 0.51)	0.659
Heneicosylic acid (21:0)	1.69 (1.51, 2.07)	1.65 (1.45, 1.90)	0.650
Behenic acid (22:0)	1.07 (0.95, 1.10)	0.90 (0.76, 1.01)	**0.005**
Lignoceric acid (24:0)	2.71 (2.19, 3.02)	2.19 (1.58, 2.83)	0.190
MUFA			
Vaccenic acid (18:1 ω-7)	0.69 (0.67, 0.83)	0.83 (0.70, 0.87)	0.264
Oleic acid (18:1 ω-9)	12.49 (12.01, 13.17)	12.39 (11.68, 13.61)	0.942
Gondoic acid (20:1 ω-9)	0.42 (0.38, 0.44)	0.42 (0.36, 0.48)	0.914
Erucic acid (22:1 ω-9)	0.49 (0.22, 0.55)	0.60 (0.30, 0.94)	0.291
Nervonic acid (24:1 ω-9)	2.45 (2.05, 2.62)	2.13 (1.81, 2.71)	0.627
PUFA			
Linoleic acid (18:2 ω-6)	8.83 (8.26, 9.51)	8.63 (7.89, 9.09)	0.332
Arachidonic acid (20:4 ω-6)	12.47 (11.36, 13.81)	12.17 (11.41, 13.73)	0.903
Eicosapentaenoic acid (20:5 ω-3)	0.43 (0.39, 0.52)	0.50 (0.43, 0.55)	0.092
Docosapentaenoic acid (22:5 ω-3)	1.68 (1.49, 1.85)	1.63 (1.52, 1.81)	0.771
Docosahexaenoic acid (22:6 ω-3)	2.78 (2.56, 3.48)	3.65 (3.31, 3.80)	**0.009**

Data are summarized as median (25th, 75th percentile). Mann– Whitney tests are used to compare sequence groups; p-values are presented for two-sided tests. Significance (α) level is set to 0.05. MUFA: mono-unsaturated fatty acid; PUFA: polyunsaturated fatty acid; SFA: saturated fatty acid

Table S2. Red blood cell (RBC) membrane fatty acid composition (%) of the participants on the conventional fish (CF) by period.

	End of 1st period	% change	End of 2nd period	% change	*p**
SFA					
Myristic acid (14:0)	0.33 (0.21, 0.50)	-16.67 (-45.45, 30.0)	0.32 (0.26, 0.36)	-29.17 (-46.15, 2.86)	0.112
Palmitic acid (16:0)	22.0 (21.05, 23.2)	-0.71 (-2.75, 1.91)	23.0 (21.89, 23.51)	-2.97 (-12.26, 1.46)	0.059
Margaric acid (17:0)	0.38 (0.36, 0.46)	-13.13 (-26.66, 1.72)	0.42 (0.36, 0.46)	-2.66 (-20.71, 25.83)	0.134
Stearic acid (18:0)	16.65 (16.18, 17.53)	-0.67 (-3.52, 1.27)	16.27 (15.43, 17.15)	-3.47 (-7.17, 3.15)	0.211
Arachidic acid (20:0)	0.63 (0.44, 0.75)	9.82 (-4.90, 49.04)	0.45 (0.37, 0.86)	22.66 (-7.14, 76.28)	**0.028**
Heneicosylic acid (21:0)	1.45 (1.14, 1.74)	-11.59 (-24.68, -4.20)	1.44 (1.16, 1.60)	-2.42 (-13.77, 4.67)	**0.044**
Behenic acid (22:0)	1.05 (0.93, 1.15)	-3.67 (-30.43, 9.09)	0.89 (0.78, 1.01)	3.06 (-26.50, 15.79)	0.477
Lignoceric acid (24:0)	2.7 (2.31, 3.0)	-1.49 (-9.35, 11.87)	2.26 (1.87, 2.5)	-2.02 (-13.27, 10.31)	0.629
MUFA					
Vaccenic acid (18:1 ω-7)	0.76 (0.68, 0.82)	9.68 (-4.35, 21.18)	0.86 (0.73, 0.91)	4.62 (-10.53, 17.74)	0.461
Oleic acid (18:1 ω-9)	12.01 (11.27, 12.97)	-0.52 (-5.11, 1.13)	12.05 (11.05, 13.58)	-3.66 (-6.94, -1.15)	0.110
Gondoic acid (20:1 ω-9)	0.49 (0.37, 0.56)	11.36 (2.78, 22.64)	0.46 (0.32, 0.62)	56.03 (-24.14, 95.83)	0.074
Erucic acid (22:1 ω-9)	0.51 (0.32, 0.74)	64.44 (-14.12, 150.0)	0.29 (0.18, 0.44)	-42.34 (-64.0, 15.38)	0.551
Nervonic acid (24:1 ω-9)	2.31 (2.28, 2.94)	-1.18 (-9.73, 11.71)	2.48 (1.97, 2.7)	6.49 (-13.41, 13.62)	0.800
PUFA					
Linoleic acid (18:2 ω-6)	8.66 (8.3, 9.2)	-1.77 (-4.84, 3.11)	8.54 (8.17, 8.93)	-2.44 (-10.15, -0.11)	0.087
Arachidonic acid (20:4 ω-6)	12.38 (11.22, 13.08)	-1.63 (-8.36, 1.53)	12.13 (11.4, 12.64)	-1.96 (-10.02, 3.79)	0.304
Eicosapentaenoic acid (20:5 ω-3)	0.58 (0.43, 0.73)	34.69 (-7.41, 86.54)	0.71 (0.58, 0.92)	37.25 (14.81, 86.36)	**<0.001**
Docosapentaenoic acid (22:5 ω-3)	1.71 (1.46, 1.8)	4.27 (-8.33, 11.11)	1.82 (1.54, 1.93)	17.31 (-3.51, 32.59)	0.154
Docosahexaenoic acid (22:6 ω-3)	3.71 (2.73, 4.2)	17.32 (-0.54, 26.40)	3.98 (3.46, 4.3)	20.94 (0.95, 30.0)	**0.001**

Data are summarized as median (25th, 75th percentile). Data are also expressed as relative change (%), calculated as the change between values at the end and values at the beginning of each treatment period. MUFA: mono-unsaturated fatty acid; PUFA: polyunsaturated fatty acid; SFA: saturated fatty acid. *Wilcoxon matched-pairs signed-rank tests were used to compare the values at the end of treatment with the values at the beginning of treatment for the combined results from the two periods; p-values are presented for two-sided tests; the significance (α) level was set to 0.05 for all tests.

Table S3. Red blood cell (RBC) membrane fatty acid composition (%) of the participants on the enriched fish (EF) by period.

	**End of 1st period**	**% change**	**End of 2nd period**	**% change**	***p****
SFA					
Myristic acid (14:0)	0.38 (0.29, 0.45)	-12.25 (-22.86, 20.83)	0.27 (0.22, 0.42)	4.66 (-26.09, 83.33)	0.969
Palmitic acid (16:0)	23.28 (21.96, 24.85)	0.68 (-4.55, 7.07)	22.0 (20.65, 22.55)	2.39 (-2.40, 4.56)	0.414
Margaric acid (17:0)	0.46 (0.37, 0.49)	-2.70 (-16.98, 6.98)	0.42 (0.32, 0.49)	-21.43 (-40.96, -4.26)	0.050
Stearic acid (18:0)	16.47 (16.12, 16.93)	-1.72 (-3.77, 0.14)	15.98 (15.71, 16.64)	-0.76 (-2.39, 0.89)	0.083
Arachidic acid (20:0)	0.56 (0.33, 0.64)	-2.5 (-30.43, 19.15)	0.43 (0.36, 0.59)	-7.69 (-41.10, 13.46)	0.627
Heneicosylic acid (21:0)	1.44 (1.24, 1.86)	-7.21 (-12.41, 0.0)	1.60 (1.29, 1.99)	-0.18 (-8.35, 3.68)	0.227
Behenic acid (22:0)	0.90 (0.77, 1.11)	6.27 (-7.81, 20.10)	1.01 (0.83, 1.11)	1.37 (-1.92, 26.25)	0.159
Lignoceric acid (24:0)	2.44 (1.84, 2.94)	6.78 (2.76, 18.31)	2.82 (2.43, 3.14)	2.01 (-5.35, 10.59)	**0.019**
MUFA					
Vaccenic acid (18:1 ω-7)	0.87 (0.78, 0.94)	11.43 (1.16, 23.21)	0.74 (0.68, 0.85)	-10.0 (-20.0, 2.41)	0.770
Oleic acid (18:1 ω-9)	11.90 (11.57, 12.91)	-2.41 (-5.76, -0.48)	11.98 (10.67, 12.8)	-2.56 (-5.61, -1.08)	**<0.001**
Gondoic acid (20:1 ω-9)	0.43 (0.34, 0.63)	-6.85 (-16.28, 32.89)	0.49 (0.30, 0.59)	0.94 (-36.17, 37.84)	0.838
Erucic acid (22:1 ω-9)	0.44 (0.32, 0.53)	11.11 (-50.0, 57.58)	0.45 (0.30, 0.79)	20.0 (-29.6, 120.0)	1.000
Nervonic acid (24:1 ω-9)	2.47 (2.14, 2.73)	14.14 (-1.62, 20.70)	2.5 (2.41, 2.69)	3.56 (-5.42, 14.56)	**0.033**
PUFA					
Linoleic acid (18:2 ω-6)	8.46 (8.02, 9.51)	0.0 (-4.37, 5.20)	8.77 (7.91, 10.05)	1.93 (-9.25, 4.04)	0.659
Arachidonic acid (20:4 ω-6)	11.30 (10.84, 12.47)	-3.79 (-8.91, -0.71)	11.19 (11.03, 12.12)	-1.61 (-10.82, -1.04)	**<0.001**
Eicosapentaenoic acid (20:5 ω-3)	0.67 (0.59, 0.79)	39.53 (9.43, 68.0)	0.76 (0.53, 0.98)	50.85 (29.27, 91.89)	**0.005**
Docosapentaenoic acid (22:5 ω-3)	1.78 (1.61, 1.91)	8.37 (-2.75, 17.74)	1.79 (1.60, 1.97)	6.21 (-17.13, 9.59)	0.338
Docosahexaenoic acid (22:6 ω-3)	4.23 (3.81, 4.65)	18.32 (13.18, 25.22)	4.02 (3.10, 4.77)	9.22 (0.91, 14.20)	**0.002**

Data are summarized as median (25th, 75th percentile). Data are also expressed as relative change (%), calculated as the change between values at the end and values at the beginning of each treatment period. MUFA: mono-unsaturated fatty acid; PUFA: polyunsaturated fatty acid; SFA: saturated fatty acid. *Wilcoxon matched-pairs signed-rank tests were used to compare the values at the end of treatment with the values at the beginning of treatment for the combined results from the two periods; p-values are presented for two-sided tests; the significance (α) level was set to 0.05 for all tests.

Table S4. Effect of the enriched fish (EF) versus the conventional fish (CF) on the activity of PAF metabolic enzymes depending on sequence (order effect).

	Mean effect (SE)	95% CI	*p*
**PAF-AH (pmol/min/mg)**			
EF (CF/EF) vs. CF (CF/EF)	0.72 (1.15)	0.55, 0.95	**0.019**
**PAF-CPT ((pmol/min/mg)**			
EF (EF/CF) vs. CF (CF/EF)	1.33 (1.14)	1.03, 1.71	**0.027**
EF (EF/CF) vs. CF (EF/CF)	1.44 (1.14)	1.12, 1.85	**0.004**

Dependent variables were ln-transformed prior to analyses due to rightly skewed distribution of data; the exposure was the treatment with fish, and covariates were the outcome evaluated at the beginning of each period, the between-subjects factor representing the two sequence groups of the study, and sex of the participants. Results are presented as the exponentiated values of the mean effect (standard error, SE) and 95% confidence interval (CI); p-values are also reported, and the significance (α) level was set to 0.05.
